# Supplementary material for: When ownership is not enough: Evaluating the co-development and uptake of the Options Assessment Toolkit (OAT) for Plasmodium vivax radical cure in malaria-endemic countries
Source: PLOS Glob Public Health. 2026 Nov 12;6(11):e0006510. doi: 10.1371/journal.pgph.0006510 (PMC13759110; doi:10.1371/journal.pgph.0006510)
Supplement: Supplementary file 1 [file pgph.0006510.s001.odt]

1
1

S1 Appendix Contents
Table A: Context of the three co-developing countries	2
Checklist A: Consolidated criteria for reporting qualitative research (COREQ) checklist: 32-item checklist	4
Checklist B: Inclusivity in global research	6
Table B: Codebook for analysis	10

# Table A: Context of the three co-developing countries

Afghanistan
Solomon Islands
Vietnam
Demographics


Population 
39.6 million
In 2018, based on revised National Statistics Office projections from the 2009 census, the estimated total national population was 667,044
In 2019, Viet Nam has an estimated population of 96 million
Malaria Epidemiology


Malaria cases confirmed 
5.6 per 1000
185.8 per 1000
0.0 per 1000
Indigenous cases
180,045
123,476
373
P. vivax cases
171,523
70,635
195
Reported deaths 
0
19
0
Imported cases 
-
-
75
Policy Context


Elimination task force in place 
Yes
No
Yes
Strategies implemented to address malaria importation
Yes
No
No 
Funding (USD)


Domestic funding (USD)
-
3,600,000
66,414,456
External funding- Global Fund (USD)
-
7,236,394
14,457,999
External other funding 
-
-
717,500
Funding gap
-
26.7%
8.5%
1 World Health Organization. World malaria report 2023. Geneva: WHO; 2023.
 World Health Organization. World malaria report 2023. Geneva: WHO; 2023.

# Checklist A: Consolidated criteria for reporting qualitative research (COREQ) checklist: 32-item checklist

Item No
Guide Questions/Description 
Reported on Page # 


Domain 1: Research team and reflexivity 


Personal Characteristics  


1. Interviewer/ facilitator 
Which author/s conducted the interview or focus group?
Pg 6


2. Credentials 
What were the researcher’s credentials? E.g., PhD, MD  
-


3. Occupation 
What was their occupation at the time of the study?
-


4. Gender 
Was the researcher male or female?
-


5. Experience and training 
What experience or training did the researcher have?
-


Relationship with participants 


6. Relationship established 
Was a relationship established prior to study commencement?
Pg 7-8


7. Participant knowledge of the interviewer  
What did the participants know about the researcher? e.g. personal goals, reasons for doing the research?  
Pg 7-8


8. Interviewer characteristics 
What characteristics were reported about the interviewer/facilitator? e.g. Bias, assumptions, reasons and interests in the research topic  
Pg 9


 Domain 2: study design 


Theoretical framework 


9. Methodological orientation and Theory  
What methodological orientation was stated to underpin the study? e.g. grounded theory, discourse analysis, ethnography, phenomenology, content analysis  
Pg 6-9


Participant selection  


10. Sampling 
How were participants selected? e.g., purposive, convenience, consecutive, snowball  
 Pg 6-7


11. Method of approach 
How were participants approached? e.g., face-to-face, telephone, mail, email  
Pg 6-7


12. Sample size 
How many participants were in the study?
Pg 6


13. Non-participation Setting 
How many people refused to participate or dropped out? Reasons?
-


14. Setting of data collection 
Where was the data collected? e.g., home, clinic, workplace  
Pg 8


15. Presence of nonparticipants 
Was anyone else present besides the participants and researchers?
-


16. Description of sample 
What are the important characteristics of the sample? e.g. demographic data, date  
 Pg 6


Data collection 

No 

17. Interview guide 
Were questions, prompts, and guides provided by the authors? Was it pilot tested?
-


18. Repeat interviews 
Were repeat interviews carried out? If yes, how many?
Pg 7-8


19. Audio/visual recording 
Did the research use audio or visual recording to collect the data?
Pg 7-8


20. Field notes 
Were field notes made during and/or after the interview or focus group? 
-


21. Duration 
What was the duration of the interviews or focus group?
Pg 7-8


22. Data saturation 
Was data saturation discussed?
Pg 8


23. Transcripts returned 
Were transcripts returned to participants for comment and/or correction?
Pg 8


Domain 3: analysis and findings


Data analysis  


24. Number of data coders 
How many data coders coded the data?
Pg 8


25. Description of the coding tree 
Did the authors provide a description of the coding tree?
-


26. Derivation of themes 
Were themes identified in advance or derived from the data?
Pg 8


27. Software 
What software, if applicable, was used to manage the data?
Pg 8


28. Participant checking 
Did participants provide feedback on the findings?
Pg 8 


Reporting  


29. Quotations presented 
Were participant quotations presented to illustrate the themes/findings? Was each quotation identified? e.g., participant number  
Pg 9-14


30. Data and findings consistent 
Was there consistency between the data presented and the findings?
-


31. Clarity of major themes 
Were major themes clearly presented in the findings?
Pg 9-14


32. Clarity of minor themes 
Is there a description of diverse cases or a discussion of minor themes?
-


# Checklist B: Inclusivity in global research

Ethical considerations, permits and authorship
This section is applicable to all research types.
Reported on page number: 8Provide details as to who granted permissions and/or consent for the study to take place in the Methods section of your manuscript. This should include the names of all ethics boards, governmental organizations, community leaders or other bodies that provided approval for the study. If individuals provided approval refer to these people by their role or title but do not list their name(s).

Reported on page number: 8
Reported on page number: No deviation reported. If there were any deviations from the study protocol after approval was obtained please provide details of these changes in the Methods section of your manuscript.

Reported on page number: No deviation reported. 
The study was conducted with NMPs of Afghanistan, the Solomon Islands, Vietnam and Nepal. These in-county officials were the participants of this study where we interviewed on experience and perceptions rather than co-investigator therefore, they are not listed as authors. 
The research team nevertheless included regional representation. Although Bijaya Shrestha holds an affiliation with Menzies School of Health Research, he is a Nepalese national living and working in Nepal, one of the study countries. Neena Valecha is an Indian national based in India and contributed additional regional expertise and contextual understanding.Did this study involve local collaborators that are residents of the country where the research was conducted or members of the community studied? If you do not have any authors from said communities, please provide an explanation for this below.  
The study was conducted with NMPs of Afghanistan, the Solomon Islands, Vietnam and Nepal. These in-county officials were the participants of this study where we interviewed on experience and perceptions rather than co-investigator therefore, they are not listed as authors. 
The research team nevertheless included regional representation. Although Bijaya Shrestha holds an affiliation with Menzies School of Health Research, he is a Nepalese national living and working in Nepal, one of the study countries. Neena Valecha is an Indian national based in India and contributed additional regional expertise and contextual understanding.

Everyone listed as an author should meet PLOS’ criteria for authorship and all individuals who meet these criteria should be included in the author byline, rather than the acknowledgements. For further information please see the journal’s Authorship Policy. 

Human subjects research (e.g. health research, medical research, cross-cultural psychology)
Verbal consent was obtained from each participant before their interview. The research was institutionally focused and the relevant community was the National Malaria Programme in each country. The participants were purposively sampled as senior technical or managerial officials directly involved in malaria programme.
The principal investigators (KT and CAL) introduced the interviewer (BS) to the co-developing NMPs, and programme leadership identified appropriate representatives. Details of participant consent are reported in the Methods.  Did you obtain written informed consent from a representative of the local community or region before the research took place? How did you establish who speaks for the community? Details of written informed consent obtained from study participants should be reported separately in the Methods section of your manuscript. 
Verbal consent was obtained from each participant before their interview. The research was institutionally focused and the relevant community was the National Malaria Programme in each country. The participants were purposively sampled as senior technical or managerial officials directly involved in malaria programme.
The principal investigators (KT and CAL) introduced the interviewer (BS) to the co-developing NMPs, and programme leadership identified appropriate representatives. Details of participant consent are reported in the Methods.  

The OAT was co-developed with the participating NMPs (Afghanistan, the Solomon Islands and Vietnam) between 2020 and 2022 through an interactive, consultative process in which documents were shared at each step and feedback was incorporated, which is evident in the result section. 
This evaluation intentionally followed up with the same participants to assess their experience of development process and use of tool. 
Transcripts were shared with participants to allow clarification or additional input. In Nepal, a non-co-developing context participants were briefed on the OAT and the questions were guided. How did members of the local community provide input on the aims of the research investigation, its methodology, and its anticipated outcome(s)? 
The OAT was co-developed with the participating NMPs (Afghanistan, the Solomon Islands and Vietnam) between 2020 and 2022 through an interactive, consultative process in which documents were shared at each step and feedback was incorporated, which is evident in the result section. 
This evaluation intentionally followed up with the same participants to assess their experience of development process and use of tool. 
Transcripts were shared with participants to allow clarification or additional input. 
In Nepal, a non-co-developing context participants were briefed on the OAT and the questions were guided. 

When engaging with the local community, how did you ensure that the informed consent documents and other materials could be understood by local stakeholders?
Participants were senior programme officials proficient in English, and the virtual interviews with participants from Afghanistan, the Solomon Islands and Vietnam were conducted in English. The interview with participants from Nepal was conducted in Nepali by BS, a native Nepali speaker, and transcribed directly into English. Transcripts were reviewed for accuracy and shared with participants so they could confirm understanding and provide clarifications.
Participants were senior programme officials proficient in English, and the virtual interviews with participants from Afghanistan, the Solomon Islands and Vietnam were conducted in English. The interview with participants from Nepal was conducted in Nepali by BS, a native Nepali speaker, and transcribed directly into English. Transcripts were reviewed for accuracy and shared with participants so they could confirm understanding and provide clarifications.

Will the findings of the research be made available in an understandable format to stakeholders in the community where the study was conducted (e.g. via a presentation, summary report, copies of publications, etc.)? Please provide details of how this will be During the study, transcripts were shared with participants for review and member checking. The findings will be disseminated through this peer-reviewed publication and have been presented to malaria control programs from the entire Asia Pacific region at the APMEN meeting in Bangkok (April 2026). achieved. 
During the study, transcripts were shared with participants for review and member checking. The findings will be disseminated through this peer-reviewed publication and have been presented to malaria control programs from the entire Asia Pacific region at the APMEN meeting in Bangkok (April 2026). 

Non-human subjects research using specimens/ animals collected as part of the study, or those housed in archival collections. Examples include archaeology, paleontology, botany and zoology. 
NADid the permission you obtained from a local authority to perform the study include an agreement on access to outputs and benefit sharing? This may include procedures to enable fair distribution of the benefits and resources arising from the research performed. Please include any details of Prior Informed Consent and Benefit Sharing Agreements obtained. These may be required by field-specific regulations, for example the Convention on Biological Diversity (CBD) and the associated Nagoya Protocol. 
NA


If the material used in your study was imported, please A) provide the year it was imported and B) indicate whether permits were obtained to import/export the materials used, C) provide details of any permits obtained. If this information is not available, please indicate this.
NA
NA


NAIf you used archival specimens, please state how the material used in your study was acquired by the institute it is held in and provide details of any permits obtained for the original excavations/ sample collection. If this information is not available, please indicate this. 
NA

NAHow was the potential cultural significance of the materials collected in your study to local communities considered in your research design? Were Indigenous peoples and/or local researchers and institutions involved with archaeological excavations / collection of specimens? If so, please provide a description of their involvement. 
NA

NAIf your manuscript includes photographs of human remains please indicate whether authors obtained permission from descendants or affiliated cultural communities to do so.
NA


# Table B: Codebook for analysis
Themes
Codes-KII
Description
Country malaria & Plasmodium vivax context
Vivax burden & trends
Statements about the burden of Plasmodium vivax (Pv) (case share, trends over time), elimination/control phase, and geographic concentration.

Case distribution & hotspots
Where cases occur (border areas, provinces/regions), seasonality, and population groups most affected (e.g., migrants).

Imported vs indigenous cases
Discussion of importation, travel history, cross-border transmission, and challenges distinguishing relapse vs reinfection.
Options Assessment Toolkit (OAT) awareness & perceived value
Awareness of OAT
Whether and how participants first learned about the Option Assessment Toolkit (OAT) and their familiarity with it.

Perceived usefulness ("thinking tool")
How OAT is described as helpful (structured framework, comprehensive picture, supports decision-making) even if not fully used.

Perceived limitations/complexity
Comments that OAT is too technical, complex, scenario-heavy, or hard for non-technical policy audiences to use.
OAT co-development process
Participation & contribution
Extent of involvement in developing OAT (reviewing drafts, meetings, providing country data/experience).

Feedback incorporation & ownership
Perceptions that suggestions were/weren't incorporated; sense of ownership and legitimacy of the final toolkit.

Mode of engagement (online vs in-person)
Experiences related to online meetings, missing face-to-face workshops, timing/frequency, and how these affected input quality.

Stakeholder representation gaps
Who was missing during development (e.g., higher-level Ministry of Health (MOH)/policymakers, provincial implementers), and why that matters.
Dissemination & uptake pathways
Dissemination channels
How OAT could/should be disseminated (World Health Organisation (WHO) channels, Asia Pacific Malaria Elimination Network {(APMEN) meetings, tech talks, workshops, national Technical Working Group (TWGs)}.

WHO endorsement as catalyst
Belief that WHO recommendation/endorsement increases credibility and adoption, especially in resource-constrained settings.

Barriers to awareness
Reasons awareness remains low (limited internet, competing priorities, weak dissemination beyond publications).
Policy change process & governance
Guideline review cycles
How often malaria guidelines are reviewed/updated and triggers for change (e.g., efficacy thresholds, WHO updates).

Decision-making actors & committees
Key decision-makers and formal bodies involved (MOH, therapeutic committees, TWGs, minister/deputy ministers).

Policy change steps & timelines
Typical steps from evidence to approval to implementation, including expected timelines and meeting cadence.

Risk aversion & regulatory hurdles
Concerns about adverse events, liability, and regulatory/import paperwork that slow or block changes (e.g., new drugs).
Financing & donor environment
Funding availability/constraints
Statements about limited funding, donor dependence, budget cuts, and how finances constrain OAT implementation and related activities.

Donor priorities & trade-offs
Examples where donors prioritize other interventions (e.g., bed nets) over Glucose-6-Phosphate Dehydrogenase (G6PD) testing, supervision, training, or pilots.

Sustainability of program inputs
Ability to maintain interventions over time (tests, reagents, supervision) when external funding declines.
Health system capacity & implementation feasibility
Human resources & turnover
Availability, skills, and retention of staff; capacity building needs; turnover due to political or workforce changes.

Service delivery model (test-treat-track)
How diagnosis, treatment, and tracking are delivered across levels {community/health posts/ Primary Health Care Centre (PHCC)/hospital}.

Logistics & supply management
Challenges with transport, distribution, stocking medicines/tests, maintaining reagents, and operating in remote areas.

Quality assurance & maintenance
Concerns about Quality Assurance / Quality Control (QA/QC), device maintenance, cold chain/materials, and competency loss when cases are rare.
G6PD testing & diagnostics
Availability of G6PD testing
Whether G6PD testing exists, at what levels, and gaps (none, pilot only, limited sites).

Type of test (qualitative/quantitative)
Discussion of test types (biosensor quantitative vs qualitative), ease of use, and appropriateness for setting.

Implementation requirements (training, QA, workflow)
Operational needs to run testing: training, supervision, QA/QC, time-to-result constraints, and referral pathways.

Cost & procurement of diagnostics
Budgeting, procurement processes, minimum order quantities, and affordability constraints for tests and consumables.
Treatment regimens & radical cure options
Primaquine (PQ) regimen selection (7/14/weekly)
Choice of PQ regimen based on G6PD status, feasibility, adherence, and guidelines (14-day, 7-day high-dose, weekly 8-week).

Tafenoquine(TQ) interest & constraints
Perceptions of TQ advantages (single-dose) and constraints (availability, approvals, controlled settings, cost, training).

Drug adherence considerations
How regimen length affects adherence; strategies to improve adherence {Directly Observed Therapy (DOT), follow-ups, reminders, incentives}.

Safety & pharmacovigilance
Adverse event concerns, monitoring capacity, reporting systems, and mitigation strategies for hemolysis risk.
Stakeholders & coordination
Key stakeholders (national & subnational)
Actors needed for uptake and implementation {MOH, (Centers for Disease Control and Prevention) CDC, institutes/hospitals, provinces, community volunteers, donors}.

Technical working groups & committees
Roles of TWGs/therapeutics committees/ethics committees in reviewing evidence and approving guideline changes.

Private sector & procurement actors
Influence (or lack) of private sector, pharmaceutical/diagnostic companies, and procurement/supply chain stakeholders.

Political engagement & advocacy
How politicians are engaged (or not), advocacy events (e.g., World Malaria Day), and implications for prioritization.
Evidence, data & research capacity
Local evidence availability
Presence/absence of local studies (G6PD prevalence, relapse, efficacy trials) and reliance on external evidence/WHO.

Efficacy vs effectiveness & relapse measurement
Discussions separating relapse vs reinfection, monitoring outcomes, and data gaps about adherence/recurrence.

Research capacity constraints
Constraints to generating local evidence (infrastructure, expertise, funding, competing workload) and suggested supports.
Recommendations for improving OAT
Simplification & usability improvements
Suggestions to simplify scenarios, add clearer decision pathways, and make language/presentation more accessible.

Training & implementation support package
Need for training of trainers, guidance notes, example workflows, and practical implementation tools to accompany OAT.

Integration into routine planning
How to embed OAT into routine policy/guideline updates, budgeting, and TWG deliberations (rather than one-off use).
OAT awareness & perceived value
OAT alignment with national guidelines
Perception that OAT overlaps with or supports existing national guidelines; OAT adds structure rather than new content.

OAT as learning tool for Pv situation
OAT used to understand the country’s Pv picture (epidemiology, system readiness) even if not used to choose a new regimen.
OAT co-development process
Scenario selection & country fit
How a country selects the most fitting OAT scenario/profile and whether it captures unique context.

Need for piloting & field validation
Calls for pilots/trials of toolkit recommendations; reflections that planned pilots were not implemented.
Dissemination & uptake pathways
Integration into TWG/working group agendas
Using OAT as an agenda item and reference framework in routine TWG meetings that update treatment guidelines.

Translation & localization needs
Need to translate OAT/training materials and tailor language/examples to local users and policy processes.
Policy change process & governance
Committee capacity & review rigor
Perceptions that approval committees are strong/weak, rubber-stamp behavior, and limited critical review due to workload/composition.

Bureaucracy & hierarchy navigation
Navigating long chains of command and paperwork for approvals (especially in centralized systems).

Efficacy study triggers (5%/10% thresholds)
Use of drug efficacy trial results and thresholds as triggers for changing first-line therapies or revising protocols.
Financing & donor environment
Domestic co-financing mechanisms
Use of domestic co-finance/budget lines to fund gaps when donors do not cover diagnostics or implementation costs.

Funding cuts affecting operations
Direct impacts of funding cuts on trainings, workshops, field monitoring, and supervision activities.
Health system capacity & implementation feasibility
Technology & connectivity barriers
Issues with internet/device use, limited connectivity, and implications for digital tools, reminders, and data reporting.

Community referral & completion
How referrals are initiated/completed (or fail) from community/CHW to facilities for radical cure or complications.

Supervision & supportive monitoring
Supervision frequency, on-the-job mentoring, and monitoring systems needed to maintain quality of testing and treatment.
G6PD testing & diagnostics
Geographic targeting of testing rollout
Strategies to implement testing first in highest-burden provinces/areas rather than nationwide scale-up.

Historical screening when testing limited
Using prior exposure/tolerability to PQ as a risk screen to cautiously scale dose changes when testing capacity is incomplete.
Treatment regimens & radical cure options
Controlled setting requirement for TQ
Discussion of what 'controlled environment' means for introducing tafenoquine (hospital-based, monitoring capacity).

TQ availability & importation process
Regulatory and supply chain steps to bring new medicines into country; reasons TQ is absent despite guideline inclusion attempts.

Patient choice & shared decision-making
Approaches that offer multiple regimens (TQ vs PQ 7 vs PQ 14) and respect patient preference when feasible.
Stakeholders & coordination
Coordination with implementing partners
Coordination dynamics with WHO/UNDP/UNICEF/PRs and how partner roles affect implementation and accountability.

Vertical program dynamics
Effects of being a well-funded vertical program: autonomy, weak ministry oversight, and reduced visibility/prioritization.
Evidence, data & research capacity
Need for compliance/adherence research
Calls to generate evidence on adherence/compliance and relapse to justify regimen changes and new drug introduction.

G6PD prevalence evidence gaps
Need for better prevalence estimates for G6PD deficiency and how uncertainty affects risk assessment and policy.
Recommendations for improving OAT
Expand toolkit scope (other species/clinical tools)
Suggestions to develop similar tools for falciparum, severe malaria, or clinical decision support for remote/frontline staff.

Add practical costing & quantification tools
Requests for simple costing, quantification, and procurement planning guidance to support decision-making and budgeting.
