# Supplementary material for: When ownership is not enough: Evaluating the co-development and uptake of the Options Assessment Toolkit (OAT) for Plasmodium vivax radical cure in malaria-endemic countries
Source: PLOS Glob Public Health. 2026 Nov 12;6(11):e0006510. doi: 10.1371/journal.pgph.0006510 (PMC13759110; doi:10.1371/journal.pgph.0006510)
Supplement: Supplementary file 2 [file pgph.0006510.s003.docx]

Journal Requirements:

1. **Please include a complete copy of PLOS’ questionnaire on inclusivity in global research in your revised manuscript. Our policy for research in this area aims to improve transparency in the reporting of research performed outside of researchers’ own country or community. The policy applies to researchers who have travelled to a different country to conduct research, research with Indigenous populations or their lands, and research on cultural artefacts. The questionnaire can also be requested at the journal’s discretion for any other submissions, even if these conditions are not met. Please find more information on the policy and a link to download a blank copy of the questionnaire here: https://journals.plos.org/globalpublichealth/s/best-practices-in-research-reporting. Please upload a completed version of your questionnaire as Supporting Information when you resubmit your manuscript.**

**Reply**: As suggested we have used the questionnaire on inclusivity and added to the supplement as table S3 Inclusivity in global research.

**2. Please provide a detailed online Financial Disclosure statement. This is published with the article. It must therefore be completed in full sentences and contain the exact wording you wish to be published.**

a) **State the initials, alongside each funding source, of each author to receive each grant. For example: “This work was supported by the National Institutes of Health (####### to AM; ###### to CJ) and the National Science Foundation (###### to AM).”**

**Reply**: We have now updated this in the manuscript and it reads as follows:

“Funding for this work was provided by Medicines for Malaria Venture (MMV), and through the Asia Pacific Leaders Malaria Alliance (APLMA) through the Asia Pacific Malaria Elimination Network (APMEN) grant. This research has been partially funded by a grant to Menzies School of Health Research from the Australian Government through the Partnerships for a Healthy Region (PHR) initiative (RESPOND). The views expressed in this publication are the author's alone and are not necessarily the views of the Australian Government. “

**b) State what role the funders took in the study. If the funders had no role in your study, please state: “The funders had no role in study design, data collection and analysis, decision to publish, or preparation of the manuscript.”**

**Reply**: We have added the statement as suggested

**3. Please ensure that the funders and grant numbers match between the Financial Disclosure field and the Funding Information tab in your submission form. Note that the funders must be provided in the same order in both places as well.**

**Reply**: We have aligned the funding information between the manuscript and the online system.

**4. Please send a completed 'Competing Interests' statement, including any COIs declared by your co-authors. Please declare all competing interests beginning with the statement "I have read the journal's policy and the authors of this manuscript have the following competing interests:**

**Reply**: We have added a competing interest statement to the end of the manuscript.

**5. We note that the ethics approval documents should not be published with the paper and are for internal information only. Please change the file type to 'Other' to ensure that they are not published.**

**Reply**: We have now changed the file type to Other.

**6. If the reviewer comments include a recommendation to cite specific previously published works, please review and evaluate these publications to determine whether they are relevant and should be cited. There is no requirement to cite these works unless the editor has indicated otherwise**.

**Reply**: No such suggestions were made by the reviewers.

**Review Comments to the Author
Reviewer #1:**

This application of a qualitative research analysis will likely be of interest to programmatic and policy audiences as well as research teams looking to support programs. Some further information around the toolkit itself and touching on options available for programs with limited resources (if possible) would help. Specifics are outlined below:

**1.Introduction – though the introduction states, “We previously developed the Options Assessment Toolkit (OAT) as a decision-support instrument in response to the first major expansion of tools for Plasmodium vivax malaria in more than six decades (3-5)” and points to references, it would be helpful if a brief explanation of what the toolkit includes and is intended to be used would be helpful.**

**Reply**: We have now added an explanation providing more detail about the OAT. This now reads as follows (lines 69-78 of the track change version):

“The OAT is a practical, evidence-based decision-support framework co-with National Malaria Programmes (NMPs) and regional experts. It was designed to help NMPs in the Asia-Pacific region select context-appropriate test-and-treat strategies for the radical cure of Plasmodium vivax malaria in their own context. It comprises five interconnected elements: a Baseline Assessment Template (14 factors across epidemiology, implementation capacity, and enabling environment) to gauge a country's readiness; eight illustrative Asia-Pacific scenarios spanning different programme phases, health systems, and politico-economic conditions; expert-recommended test-and-treat combinations; an approaches tool to help map the policy-change process and plan implementation; and a step-by-step user guide.”

**2. Results – similar to the point above – it would be helpful to have some examples of what the OAT is expected to be used for. What are some examples of potential policy adjustments that would be made when a program uses it.**

**Reply**: Please see reply to the previous comment.

**3.Results – the OAT serving as a checklist. It is hard to know how to interpret some of the responses. Did the NMP from Afghanistan mean that it points out what is missing from their available commodities? Or processes?**

**Reply**: In the interview, the NMP’s response referred to both commodities and implementation processes. The OAT helped the programme systematically assess the requirements for introducing shorter-course radical cure. This process highlighted the absence of quantitative G6PD testing as the immediate commodity gap, while also identifying related requirements, including financing, procurement, staff training, treatment-guideline revision, approval processes, and phased implementation in high-burden provinces.

We have clarified the text to show that “checklist” refers to a structured review of implementation requirements rather than merely a list of available commodities. It now reads as follows (line 215-218 of the track change version):

“Furthermore, the OAT was used as a structured checklist or planning aid, guiding programme teams to systematically review key operational requirements; such as referral systems, treatment adherence, pharmacovigilance, workforce capacity, budgets, and political support so that important considerations were not overlooked.”

**4.Results – the Vietnam NMP said that it is not that different from what they already do? Is it possible to briefly summarize what the Vietnam program does?**

**Reply**: We have added in line 226-231.

“The Vietnam participant indicated that several elements of the OAT were in the country’s national malaria guidelines and routine decision-making processes. The interview suggests that the programme periodically reviews its treatment guidelines, considers WHO recommendations and local evidence, consults relevant national stakeholders, and assesses epidemiology, treatment options, G6PD testing, patient follow-up, regulatory requirements and implementation feasibility. “

However, the interview did not provide a sufficiently detailed or formally verified account of the programme’s complete process. A formal comparison was outside of the scope of this work.

**5.Results – lines 215-217 were unclear.**

**Reply**: We have now revised these lines for clarity. It now reads as follows (line 240-246 of the track chang version):

“A central finding was that participants assessed the value of the OAT primarily through the feasibility of implementing its recommended treatment options within their existing health systems, rather than evaluating the decision-support framework itself. Consequently, perceptions of the OAT were strongly shaped by local implementation capacity and health system readiness.”

**6.Results – lines 225-226 – could OAT be adapted to provide a “next best thing” in the absence of system readiness? Like a decision tree based on the robustness of the system? From the discussion, my guess is probably not, but would be curious if this was a topic in the toolkit development process at least.**

**Reply**: As clarified in our expanded description of the OAT (line 69-78), it was not designed as a prescriptive decision tree that automatically determines the “next best” option when full system readiness is absent.

**7. Results– lines 227-229 – was it discussed that identifying gaps was a useful product in and of itself from using the toolkit?**

**Reply**: Yes, the data indicate that identifying gaps was considered a useful outcome of using the OAT, even when immediate implementation was not feasible.

**8.Results – can any more details of the results or responses be shared other than those highlighted?**

**Reply**: While additional examples could provide further context, we have prioritised the findings most directly relevant to the study objectives.

**9.Discussion – great points here. The second paragraph really resonated.**

**Reply**: We thank the reviewer for this assessment and have not made any changes to this section of the manuscript.

**10. Discussion – I agree with what you say in line 315. It is likely that the process of developing the toolkit impacted programmatic operations or at the very least mindsets. Were there questions about policy changes after OAT that may have been indirectly related to the development of OAT? Meaning: were there changes that just ‘happened’ to overlap with the post-implementation or OAT introduction timeline?**

**Reply**: While our findings suggest that the OAT's contribution was primarily conceptual rather than instrumental, our study was not designed to determine whether any broader changes in programme thinking or decision-making were attributable to the OAT or would have occurred irrespective of its introduction as part of routine programme evolution or other external influences. We have therefore clarified this point in the Discussion (line 341-351 track change version) which reads as follows:

“This variability suggests that decision-support tools cannot be uniformly designed or implemented, but must account for differing levels of analytic capacity, regulatory maturity, and institutional autonomy. Evaluating decision-support tools solely on immediate policy shifts may therefore underestimate their impact (27, 28). In complex health systems, incremental shifts in how evidence is interpreted and discussed likely represent meaningful progress. Our study was not designed to determine whether such subtle changes occurred as a result of the OAT or would have emerged through routine programme evolution and broader changes in the policy environment. For sustained influence, such tools must likely be embedded within formal policy and planning cycles, including mid-term programme reviews, NSP revisions, and associated budgeting and implementation processes.”

**11.Discussion – lines 359-361 – this is also a great point and programs having the ‘confidence’ or ability to deviate from simply what is prescribed by WHO or otherwise could results in innovative strategies.**

**Reply**: We thank the reviewer for this assessment and have not made any changes to this section of the manuscript.

**Reviewer #2:** This manuscript by Shrestha et al. addresses an important and timely topic by qualitatively assessing users' experiences with the co-development and early implementation of the Options Assessment Toolkit (OAT), which is intended to support National Malaria Control Programmes (NMCPs) in selecting the most appropriate Plasmodium vivax radical cure strategies. Understanding the utility and practicality of the OAT among its intended users and policymakers across different country contexts (i.e. co-developing and non-co-developing countries) could potentially facilitate the adoption of appropriate P. vivax radical cure policies. Overall, the manuscript is well written and presents valuable insights. However, I have several minor comments for the authors to consider.

**1.Line 66: Typographical error noted ("...co-developed between 2020 and 2022 as...").**

**Reply**: Corrected

**2.Line 91: It is understandable that three countries (Afghanistan, the Solomon Islands, and Vietnam) were involved as the original co-developing countries for the OAT. However, I wonder why only one additional country, Nepal (outside the original co-developing settings and represented by only two participants), was selected from the 22 official member states in the Asia Pacific region. Does the inclusion of only one non-co-developing country provide sufficient basis for an unbiased comparison of "...between co-development-driven ownership and independent engagement with the toolkit" (Lines 92–93)?**

**Reply**: We agree that the inclusion of a single non-co-developing country does not provide a sufficient basis for a formal comparison between co-development-driven ownership and independent engagement with the OAT. Our intention was not to undertake a full comparative analysis, but rather to explore whether perspectives from outside the original co-development settings aligned with or differed from those of the co-developing countries. We have revised the text to clarify this now reads as follows (line 102-106 track change version):

“In addition, we included participants from Nepal to explore how the tool was perceived and used outside the original co-developing settings. Although limited to one country, this provided an opportunity for comparison between co-development-driven ownership and independent engagement with the toolkit; it was not intended to support a formal comparison between countries.”

**3.Lines 95–96: The authors state that participants held "...senior technical or managerial roles within the NMCPs and were directly involved in malaria policy formulation or advisory processes." Could the authors provide additional information regarding the participants' experience in these positions? For example, how many years had they served in these roles?**

**Reply**: Participants held senior technical or managerial responsibilities within their respective NMCPs and were purposively selected because of their direct involvement in malaria guideline development, policy advice, programme implementation, and/or co-development of the OAT. We did not add further information to the manuscript as additional detail could identify participants.

**4.Lines 105–107: The study included a total of nine participants from the original co-developing settings and only two participants from a non-co-developing setting. Please clarify how participants were selected. Was purposive sampling employed? Was the sample size enough? Were there any inclusion or exclusion criteria? Did any eligible participants decline to participate?**

**Reply**: We have revised the respective section in the methods section to provide more clarity. This now reads as follows (line 96-11):

“This study employed a qualitative approach involving the original co-developing countries; Afghanistan, the Solomon Islands and Vietnam (Table S1) to explore their experience of the OAT development process. Participants in these countries were NMCP representatives who had been directly involved in the co-development of the OAT. The evaluation therefore intentionally followed up with the same institutional actors to assess their experiences of the development process and subsequent use of the tool.

In addition, we included participants from Nepal to explore how the tool was perceived and used outside the original co-developing settings. Although limited to one non-co-developing country, this provided an opportunity for comparison between co-development-driven ownership and independent engagement with the toolkit; it was not intended to support a formal comparison between countries.

Given the institutional focus of the study, participants were purposively sampled as individuals occupying senior technical or managerial roles within the NMCPs and directly involved in malaria policy formulation or advisory processes. The aim was not numerical representativeness but to capture perspectives from actors positioned within national decision-making structures. “

**5.Lines 261–267: The manuscript states that "Nepal participants described a different perspective." I wonder whether this observation reflects the fact that Nepal was not originally involved in the co-development of the OAT. Consequently, their views may represent a more independent assessment of the toolkit, without any perceived ownership or prior investment in its development.**

**Reply**: We agree that Nepal's different relationship with the OAT may have influenced participants' perspectives. Indeed, one of the reasons for including Nepal was to explore perceptions from a setting that had not been involved in the original co-development of the toolkit. However we felt it would be inappropriate to speculate on the reasons for these differences in the Results section and have therefore not made any changes in this section.

**6.Line 297: Typographical error noted ("...sstructured...").**

**Reply**: We have corrected this typo.

**General comments:

7. Could the authors elaborate on what additional steps are required to ensure that the OAT will be widely accepted and adopted as a decision-support tool by NMCPs?**

**Reply**: Based on the results presented we don’t think the current version of the OAT will be widely accepted and used. We have revised in line 349-351 (track change version):

“Future iterations of this tool must likely be embedded within formal policy and planning cycles, including mid-term programme reviews, NSP revisions, and associated budgeting and implementation process”

**8.WHO guidelines are developed through extensive consultation with international experts and are based on broad global consensus, whereas the OAT appears to have been developed from a limited number of country settings. Could the authors explain why the OAT has been developed as a standalone decision-support tool rather than being incorporated into existing WHO guidance? Furthermore, how do the authors envision the relationship between the OAT and future WHO recommendations for P. vivax radical cure policies?**

**Reply**: The OAT was not developed as an alternative to WHO guidance, nor was it intended to establish independent treatment recommendations. Rather, it was developed on the premise that WHO recommendations are necessarily global in scope and therefore cannot account for the diversity of epidemiological, health system, operational, and financial contexts across countries. Specially, the OAT was developed in anticipation of a policy landscape in which multiple WHO-endorsed radical cure options would become available. Its purpose was to support countries in determining which of these WHO-endorsed options would be most appropriate for their specific context, taking into account factors such as transmission patterns, G6PD testing capacity, health system readiness, implementation feasibility, and resource availability.

The framework was initially co-developed with a small number of countries to ensure that it addressed the practical needs of national malaria programmes. This evaluation was not designed to assess whether the OAT should be incorporated into WHO guidance, and this question lies beyond the scope of the present study. However, we envisage decision-support tools such as the OAT as complementing WHO normative guidance by facilitating country-level adaptation and implementation, rather than replacing or competing with WHO recommendations.

We have highlighted this in the manuscript in lines 376-392.
