# Supplementary material for: When ownership is not enough: Evaluating the co-development and uptake of the Options Assessment Toolkit (OAT) for Plasmodium vivax radical cure in malaria-endemic countries
Source: PLOS Glob Public Health. 2026 Nov 12;6(11):e0006510. doi: 10.1371/journal.pgph.0006510 (PMC13759110; doi:10.1371/journal.pgph.0006510)
Supplement: Peer review history

# Peer review: Decision Letter 0

**DOI:** 10.1371/journal.pgph.0006510.r001

**Authors:** Emma Campbell

12 Jul 2026

PGPH-D-26-01642

When ownership is not enough: Evaluating the co-development and uptake of the Options Assessment Toolkit (OAT) for Plasmodium vivax radical cure in malaria-endemic countries

PLOS Global Public Health

Dear Dr. Thriemer,

Thank you for submitting your manuscript to PLOS Global Public Health. After careful consideration, we feel that it has merit but does not fully meet PLOS Global Public Health’s publication criteria as it currently stands. Therefore, we invite you to submit a revised version of the manuscript that addresses the points raised during the review process.

The manuscript has been assessed by two reviewers and their comments can be found below. Please review their reports ad make the appropriate revisions to address all concerns raised.

Please submit your revised manuscript by Sep 08 2026 11:59PM. If you will need more time than this to complete your revisions, please reply to this message or contact the journal office at globalpubhealth@plos.org. When you're ready to submit your revision, log on to https://www.editorialmanager.com/pgph/ and select the 'Submissions Needing Revision' folder to locate your manuscript file.

Please include the following items when submitting your revised manuscript:

- A letter that responds to each point raised by the editor and reviewer(s). You should upload this letter as a separate file labeled 'Response to Reviewers'.
- A marked-up copy of your manuscript that highlights changes made to the original version. You should upload this as a separate file labeled 'Revised Manuscript with Track Changes'.
- An unmarked version of your revised paper without tracked changes. You should upload this as a separate file labeled 'Manuscript'.

Guidelines for resubmitting your figure files are available below the reviewer comments at the end of this letter.

As the corresponding author, your ORCID iD is verified in the submission system and will appear in the published article. PLOS supports the use of ORCID, and we encourage all coauthors to register for an ORCID iD and use it as well. Please encourage your coauthors to verify their ORCID iD within the submission system before final acceptance, as unverified ORCID iDs will not appear in the published article. *Only*  the individual author can complete the verification step; PLOS staff *cannot*  verify ORCID iDs on behalf of authors.

We look forward to receiving your revised manuscript.

Kind regards,

Emma Campbell, Ph.D

Staff Editor

PLOS Global Public Health

Journal Requirements:

1. Please include a complete copy of PLOS’ questionnaire on inclusivity in global research in your revised manuscript. Our policy for research in this area aims to improve transparency in the reporting of research performed outside of researchers’ own country or community. The policy applies to researchers who have travelled to a different country to conduct research, research with Indigenous populations or their lands, and research on cultural artefacts. The questionnaire can also be requested at the journal’s discretion for any other submissions, even if these conditions are not met. Please find more information on the policy and a link to download a blank copy of the questionnaire here: https://journals.plos.org/globalpublichealth/s/best-practices-in-research-reporting. Please upload a completed version of your questionnaire as Supporting Information when you resubmit your manuscript.

2. Please provide a detailed online Financial Disclosure statement. This is published with the article. It must therefore be completed in full sentences and contain the exact wording you wish to be published.

a) State the initials, alongside each funding source, of each author to receive each grant. For example: “This work was supported by the National Institutes of Health (####### to AM; ###### to CJ) and the National Science Foundation (###### to AM).”

b) State what role the funders took in the study. If the funders had no role in your study, please state: “The funders had no role in study design, data collection and analysis, decision to publish, or preparation of the manuscript.”

For more information, please go to our submission guidelines:

https://journals.plos.org/globalpublichealth/s/submission-guidelines#loc-financial-disclosure-statement

3. Please ensure that the funders and grant numbers match between the Financial Disclosure field and the Funding Information tab in your submission form. Note that the funders must be provided in the same order in both places as well.

4. Please send a completed 'Competing Interests' statement, including any COIs declared by your co-authors. Please declare all competing interests beginning with the statement "I have read the journal's policy and the authors of this manuscript have the following competing interests:

For more information, please go to our submission guidelines:

https://journals.plos.org/globalpublichealth/s/submission-guidelines#loc-competing-interests

5. We note that the ethics approval documents should not be published with the paper and are for internal information only. Please change the file type to 'Other' to ensure that they are not published.

6. If the reviewer comments include a recommendation to cite specific previously published works, please review and evaluate these publications to determine whether they are relevant and should be cited. There is no requirement to cite these works unless the editor has indicated otherwise.

Additional Editor Comments (if provided):

[Note: HTML markup is below. Please do not edit.]

Reviewers' comments:

Reviewer's Responses to Questions

**Comments to the Author**

1. Does this manuscript meet PLOS Global Public Health’s publication criteria?

Reviewer #1: Yes

Reviewer #2: Yes

**********

2. Has the statistical analysis been performed appropriately and rigorously?

Reviewer #1: N/A

Reviewer #2: N/A

**********

3. Have the authors made all data underlying the findings in their manuscript fully available (please refer to the Data Availability Statement at the start of the manuscript PDF file)?

The PLOS Data policy

Reviewer #1: No

Reviewer #2: Yes

**********

4. Is the manuscript presented in an intelligible fashion and written in standard English?

Reviewer #1: Yes

Reviewer #2: Yes

**********

Reviewer #1: This application of a qualitative research analysis will likely be of interest to programmatic and policy audiences as well as research teams looking to support programs. Some further information around the toolkit itself and touching on options available for programs with limited resources (if possible) would help. Specifics are outlined below:

1. Introduction – though the introduction states, “We previously developed the Options Assessment Toolkit (OAT) as a decision-support instrument in response to the first major expansion of tools for Plasmodium vivax malaria in more than six decades (3-5)” and points to references, it would be helpful if a brief explanation of what the toolkit includes and is intended to be used would be helpful.

2. Results – similar to the point above – it would be helpful to have some examples of what the OAT is expected to be used for. What are some examples of potential policy adjustments that would be made when a program uses it.

3. Results – the OAT serving as a checklist. It is hard to know how to interpret some of the responses. Did the NMP from Afghanistan mean that it points out what is missing from their available commodities? Or processes?

4. Results – the Vietnam NMP said that it is not that different from what they already do? Is it possible to briefly summarize what the Vietnam program does?

5. Results – lines 215-217 were unclear.

6. Results – lines 225-226 – could OAT be adapted to provide a “next best thing” in the absence of system readiness? Like a decision tree based on the robustness of the system? From the discussion, my guess is probably not, but would be curious if this was a topic in the toolkit development process at least.

7. Results – lines 227-229 – was it discussed that identifying gaps was a useful product in and of itself from using the toolkit?

8. Results – can any more details of the results or responses be shared other than those highlighted?

9. Discussion – great points here. The second paragraph really resonated.

10. Discussion – I agree with what you say in line 315. It is likely that the process of developing the toolkit impacted programmatic operations or at the very least mindsets. Were there questions about policy changes after OAT that may have been indirectly related to the development of OAT? Meaning: were there changes that just ‘happened’ to overlap with the post-implementation or OAT introduction timeline?

11. Discussion – lines 359-361 – this is also a great point and programs having the ‘confidence’ or ability to deviate from simply what is prescribed by WHO or otherwise could results in innovative strategies.

Reviewer #2: This manuscript by Shrestha et al. addresses an important and timely topic by qualitatively assessing users' experiences with the co-development and early implementation of the Options Assessment Toolkit (OAT), which is intended to support National Malaria Control Programmes (NMCPs) in selecting the most appropriate Plasmodium vivax radical cure strategies. Understanding the utility and practicality of the OAT among its intended users and policymakers across different country contexts (i.e. co-developing and non-co-developing countries) could potentially facilitate the adoption of appropriate P. vivax radical cure policies. Overall, the manuscript is well written and presents valuable insights. However, I have several minor comments for the authors to consider.

Line 66: Typographical error noted ("...co-developed between 2020 and 2022 as...").

Line 91: It is understandable that three countries (Afghanistan, the Solomon Islands, and Vietnam) were involved as the original co-developing countries for the OAT. However, I wonder why only one additional country, Nepal (outside the original co-developing settings and represented by only two participants), was selected from the 22 official member states in the Asia Pacific region. Does the inclusion of only one non-co-developing country provide sufficient basis for an unbiased comparison of "...between co-development-driven ownership and independent engagement with the toolkit" (Lines 92–93)?

Lines 95–96: The authors state that participants held "...senior technical or managerial roles within the NMCPs and were directly involved in malaria policy formulation or advisory processes." Could the authors provide additional information regarding the participants' experience in these positions? For example, how many years had they served in these roles?

Lines 105–107: The study included a total of nine participants from the original co-developing settings and only two participants from a non-co-developing setting. Please clarify how participants were selected. Was purposive sampling employed? Was the sample size enough? Were there any inclusion or exclusion criteria? Did any eligible participants decline to participate?

Lines 261–267: The manuscript states that "Nepal participants described a different perspective." I wonder whether this observation reflects the fact that Nepal was not originally involved in the co-development of the OAT. Consequently, their views may represent a more independent assessment of the toolkit, without any perceived ownership or prior investment in its development.

Line 297: Typographical error noted ("...sstructured...").

General comments:

Could the authors elaborate on what additional steps are required to ensure that the OAT will be widely accepted and adopted as a decision-support tool by NMCPs?

WHO guidelines are developed through extensive consultation with international experts and are based on broad global consensus, whereas the OAT appears to have been developed from a limited number of country settings. Could the authors explain why the OAT has been developed as a standalone decision-support tool rather than being incorporated into existing WHO guidance? Furthermore, how do the authors envision the relationship between the OAT and future WHO recommendations for P. vivax radical cure policies?

**********

what does this mean?). If published, this will include your full peer review and any attached files.

**Do you want your identity to be public for this peer review?**  If you choose “no”, your identity will remain anonymous but your review may still be made public.

For information about this choice, including consent withdrawal, please see our Privacy Policy

Reviewer #1: No

Reviewer #2: No

**********

[NOTE: If reviewer comments were submitted as an attachment file, they will be attached to this email and accessible via the submission site. Please log into your account, locate the manuscript record, and check for the action link "View Attachments". If this link does not appear, there are no attachment files.]

# Author comment: Author response to Decision Letter 1

**DOI:** 10.1371/journal.pgph.0006510.r002

31 Jul 2026

Attachment Submitted filename: OAT evaluation_ point by point response.docx

# Peer review: Decision Letter 1

**DOI:** 10.1371/journal.pgph.0006510.r003

**Authors:** Julia Robinson

28 Oct 2026

When ownership is not enough: Evaluating the co-development and uptake of the Options Assessment Toolkit (OAT) for Plasmodium vivax radical cure in malaria-endemic countries

PGPH-D-26-01642R1

Dear Dr. Thriemer,

We are pleased to inform you that your manuscript 'When ownership is not enough: Evaluating the co-development and uptake of the Options Assessment Toolkit (OAT) for Plasmodium vivax radical cure in malaria-endemic countries' has been provisionally accepted for publication in PLOS Global Public Health.

Before your manuscript can be formally accepted you will need to complete some formatting changes, which you will receive in a follow up email. A member of our team will be in touch with a set of requests.

Please note that your manuscript will not be scheduled for publication until you have made the required changes, so a swift response is appreciated.

IMPORTANT: The editorial review process is now complete. PLOS will only permit corrections to spelling, formatting or significant scientific errors from this point onwards. Requests for major changes, or any which affect the scientific understanding of your work, will cause delays to the publication date of your manuscript.

If your institution or institutions have a press office, please notify them about your upcoming paper to help maximize its impact. If they'll be preparing press materials, please inform our press team as soon as possible -- no later than 48 hours after receiving the formal acceptance. Your manuscript will remain under strict press embargo until 2 pm Eastern Time on the date of publication. For more information, please contact globalpubhealth@plos.org.

Thank you again for supporting Open Access publishing; we are looking forward to publishing your work in PLOS Global Public Health.

Best regards,

Julia Robinson

Executive Editor

PLOS Global Public Health

***********************************************************

Reviewer Comments (if any, and for reference):

Reviewer's Responses to Questions

**Comments to the Author**

Reviewer #3: All comments have been addressed

**********

publication criteria?

Reviewer #3: Yes

**********

3. Has the statistical analysis been performed appropriately and rigorously?

Reviewer #3: N/A

**********

4. Have the authors made all data underlying the findings in their manuscript fully available (please refer to the Data Availability Statement at the start of the manuscript PDF file)?

The PLOS Data policy

Reviewer #3: Yes

**********

5. Is the manuscript presented in an intelligible fashion and written in standard English?

Reviewer #3: Yes

**********

Reviewer #3: This is the second review on the manuscript entitled "When ownership is not enough: Evaluating the co-development and uptake of the Options Assessment Toolkit (OAT) for Plasmodium vivax radical cure in malaria-endemic ountries".

The research's findings are relevant for anyone providing or receiving technical guidance on decision-making in national disease control strategies. The ttile

The revised manuscript address the reviewer's comments adequately and reads well and clear.

I have no further comments or questions that would require further revision.

**********

what does this mean?). If published, this will include your full peer review and any attached files.

**Do you want your identity to be public for this peer review?**  If you choose “no”, your identity will remain anonymous but your review may still be made public.

For information about this choice, including consent withdrawal, please see our Privacy Policy

Reviewer #3: No

**********
